# Paramutation: Just a Curiosity or Fine Tuning of Gene Expression in the Next Generation?

**DOI:** 10.2174/138920211795860099

**Published:** 2011-06

**Authors:** Roberto Pilu

**Affiliations:** Dipartimento di Produzione Vegetale, Università degli Studi di Milano, Via Celoria 2, 20133 Milano, Italy

**Keywords:** Epigenetics, DNA methylation, gene silencing, paramutation, repeated sequences, RNA-directed transcriptional silencing.

## Abstract

Gene silencing is associated with heritable changes in gene expression which occur without changes in DNA sequence. In eukaryotes these phenomena are common and control important processes, such as development, imprinting, viral and transposon sequence silencing, as well as transgene silencing. Among the epigenetic events, paramutation occurs when a silenced allele (named paramutagenic) is able to silence another allele (paramutable) in trans and this change is heritable. The silenced paramutable allele acquires paramutagenic capacity in the next generations. In the 1950s, Alexander Brink described for the first time the phenomenon of paramutation, occurring in maize at the *colored1* (*r1*) gene, a complex locus (encoding myc-homologous transcription factors) that regulates the anthocyanin biosynthetic pathway. Since then, paramutation and paramutation-like interactions have been discovered in other plants and animals, suggesting that they may underlie important mechanisms for gene expression. The molecular bases of these phenomena are unknown. However in some cases, the event of paramutation has been correlated with changes in DNA methylation, chromatin structure and recently several studies suggest that RNA could play a fundamental role. This last consideration is greatly supported by genetic screening for mutants inhibiting paramutation, which allowed the identification of genes involved in RNA-directed transcriptional silencing, although it is possible that proteins are also required for paramutation.

The meaning of paramutation in the life cycle and in evolution remains to be determined even though we might conjecture that this phenomenon could be involved in a fast heritability of favourable epigenetic states across generations in a non-Mendelian way.

## PARAMUTATION: A PARTICULAR EPIGENETIC PHENOMENON

Cell specialization in complex organisms is possible by fine tuning of genome expression because all the cells of a multicellular organism carry the same DNA information but only a small sub-set of the genes must be active at a certain point of development and growth [1]. Furthermore this “molecular memory” regarding the level of expression of every gene must be faithfully transmitted through cell division while also allowing the adaptation of the organism to the environmental stimuli during its life.

Since Mendel’s laws were rediscovered a huge amount of work has been done confirming the universality of these findings: nevertheless some exceptions were identified from several studies done by earlier geneticists. In the 1940s Barbara McClintock was one of the first scientists to find exceptions to Mendel’s laws while working on anthocyanin pigments in maize: this work led to the discovery of transposons and to the Nobel prize in 1983 [2, 3].

In particular the epigenetic phenomena defined by Riggs and colleagues as “the study of mitotically and/or meiotically heritable changes in gene function that cannot be explained by changes in DNA sequence” have disclosed a new level of gene regulation [4].

These phenomena seem as if they may exist in all phyla, and control a number of gene regulation processes ranging from embryo development to human diseases by DNA methylation, chromatin modification (histone methylation and nucleosome position) and noncoding RNA [5-7].

Paramutation is an epigenetic phenomenon in which an epigenetic state of an allele (named paramutagenic) is transferred to another allele (paramutable) in trans, resulting in a heritable modification of its gene expression (the frequency of the change can reach as high as 100%), the paramutable allele acquires the paramutagenic capability in future generations, while alleles that do not take part in paramutation are nominated as neutral Fig. (**1**). Differently from a typical mutation, in which the change of the DNA sequence usually causes a switch off of the gene activity, paramutation generates different epialleles silenced with variable phenotypes due to modulation of gene expression, leading to a change in the conception of gene expression from a digital system (the classical mutations) to an analog system (epialleles) [8].

Although classical paramutation was well defined by the maize model, in the past, several gene silencing/ paramutation-like phenomena have been discovered in all the kingdoms of eukaryotes, leading to the adoption of different names such as: transvection in Drosophila [9], co-suppression and “virus-induced gene silencing" (VIGS) in the gene silencing phenomena described in transgenic plants [10-12], quelling in the fungus *Neurospora crassa* [11] and RNA interference (RNAi) in the nematode *C. elegans* [12]. This last discovery was made by Graig C. Mello and Andrew Fire: they demonstrated that double-stranded RNA injected into *C. elegans* silenced the endogenous targeted gene, and for the clinical therapy potential of this technique they won the Nobel prize in 2006.

## PARAMUTATION IN PLANTS

So far, classical paramutation in plants has been noticed in maize at five loci: *colored 1* (*r1*), *booster 1* (*b1*), *purple plant 1* (*pl1*), *pericarp color 1* (*p1*) and *low phytic acid 1* (*lpa1*) [13] and in tomato at the *sulfurea* (*sulf*) locus [14]. In maize the *r1*, *b1*, *pl1* and *p1* genes encode all for transcription factors involved in the regulation of accumulation of flavonoids and anthocyanins in several plant tissues [15] while *lpa1* locus designated *ZmMRP4*, coding for a multidrug resistance-associated-protein, is involved in phytic acid transport and storage in the seed [16,17]. In 1956, Alexander Brink, also working on anthocyanin biosynthesis, first discovered in maize a paramutation phenomenon in a regulatory gene (encoding myc-homologous transcription factors) named *colored1* (*r1*) [18]. When Brink crossed the paramutagenic *R-stippled* (*R-st*), conferring tiny spotted aleurone colour of the seed, with the paramutable allele *R-r*, conferring full pigmentation, he observed in the progeny carrying *R-r* allele a variably reduced pigmentation. The silenced allele (named *R-r’*) was heritable and capable of weak paramutagenic activity (like *R-st*) for some generations, furthermore *R-r’* reverted to *R-r* normal phenotype in few generations if it was not further exposed to *R-st* Fig. (**2A**) [19].

In the case of *b1*, the paramutable *B-I* (*Booster-Intense*) allele spontaneously becomes partially silent (this “new allele” is coded *B’*) with a frequency ranging from 0.1 to 10%. *B’* has paramutagenic activity, in fact crossing *B’* with *B-I* the progeny obtained is 100% *B’* Fig. (**2B**) [20, 21]. In contrast with *r1* paramutation *B’* is permanent; in point of fact no changes to *B-I* have been observed over tens of years and thousands of plants [21]. In the 1990s, paramutation was discovered at (*pl1*) locus, also in this case, the exposure in trans of paramutable allele *Pl-Rhoades* (*Pl-Rh*) to its spontaneously derived silenced paramutagenic *Pl’* allele causes a silencing of *Pl-Rh* Fig. (**3**) [22]. In the case of the *p1* locus, the spontaneously silenced epiallele (*P-rr’*) showed a moderate stability and weak paramutagenic capacity on the original *P-rr* allele [23], furthermore, the paramutagenic *P-rr’* silenced allele arises by transgene induced silencing [24]. Interestingly, some differences among these paramutation systems can be noted: *p1* and *r1* epigenetic states are stable while *pl1* and *b1* loci are unstable, in fact they spontaneously change to the silenced state with high frequency [15, 24]. Recently in maize a new locus undergoing a paramutation phenomenon has been discovered which does not involve the anthocyanin pathway: the *lpa1-241* allele at the *lpa1* locus in fact seems somewhat similar to *r1* locus paramutation [17]. The *lpa1-241* mutant (originally isolated from a chemically mutagenized populations using EMS) does not modify the total amount of seed phosphorous, but reduces phytic acid content correlated to a proportionally increased level of free phosphate associated to severe negative pleiotropic effects, therefore the mutation is propagated as heterozygous [25,  26]. Also in this case the *lpa1-241* paramutagenic allele is able to partially silence the paramutable *Lpa1* allele when exposed in trans and this effect is strengthen by the progressive exposure to the paramutagenic allele in the next generations [17].

The last case treated in this review of classical paramutation in plants was observed in tomato at the locus *sulfurea* (*sulf*) isolated by R. Hagemann in 1958 after an X-ray mutagenesis experiment [27]. The recessive *sulfurea* mutant showed a chlorophyll-deficient phenotype (sulfurous colour) and even though so far this gene was not isolated it seems likely that this phenotype is caused by an auxin deficiency [28]. The *sulf* homozygous plants do not survive beyond the seedling stage, thus paramutation at the tomato sulfurea pigment deficiency appeared at high frequency as somatic sectors in *Sulf/sulf* heterozygous plants Fig. (**2C**). The seeds obtained from sulf sector (where the *sulf* allele has paramutated the + *Sulf* allele) are all *sulf/sulf* whilst the seeds obtained from the green sectors produce again plants with *sulf* somatic sectors [27, 28]. The level of paramutagenicity of different paramutagenic alleles is different, in fact in the case of *B’* and *Pl’* alleles it is strong [22], while for *R-st* [29], *P-rr’* [24, 30] and *lpa1-241* [17] alleles it is variable.

In all these cases of paramutation (with the exception of the *sulf* locus where so far the corresponding gene has not been isolated) it has been demonstrated that paramutated alleles correlate with a reduction of mRNA levels [15, 17, 23, 24, 31-36].

## PARAMUTATION IN ANIMALS

In animals, gene silencing phenomena have been well studied in different cases such as somatic inactivation of the mammalian X chromosome [37] and in general in the transcriptional silencing of heterochromatin regions of the genome [38]. However for several years, among the epigenetic phenomena, paramutation has been considered as an odd or peculiar plant-linked event involving either partial or total gene silencing. In recent years some cases of paramutation–like phenomena have been discovered by studying the mouse (*Mus musculus*) model system: the *Rosa26* locus [39], the *Rasgrf1* locus [40] and the *Kit* locus [41], all arose by modifying the genes sequence using transgenic techniques and the *Agouti viable yellow* (*A^vy^*) allele was produced by a retrotransposon insertion close to the promoter region [42].

The *Kit* locus (*Kit* gene encodes for the receptor tyrosine kinase) is the best studied case of animal paramutation. The *Kit^tm1Alf^* produced by insertional mutagenesis is a null allele lethal in the homozygous state, the viable heterozygous  mice (*Kit^tm1Alf^* /+) have white tail tips (and white feet) in contrast with the wild types (+/+) that have coloured tail tips. When heterozygous mice (*Kit^tm1Alf^* /+) were crossed to wild type the progeny obtained were all phenotypically identical to their heterozygous parent having the white tail tips, in contrast with the expected 1 *Kit^tm1Alf^* /+ : 1 +/+ Mendelian segregation ratio Fig. (**2D**). This means that the + paramutable alleles are paramutated by exposure to the paramutagenic *Kit^tm1Alf^* allele, furthermore the +/+ paramutated mice with white tail tips named Kit* can transmit this phenotype to the future generations although with a reduced penetrance [41] as observed for example in the case of paramutation of *b1* gene in maize [21].

There is also some evidence that paramutation-like phenomena in humans could be involved in diseases such as *insulin-dependent diabetes mellitus 2* (*IDDM2*) [43], cancer [44], miR-1-induced cardiac hypertrophy [45] and the paternal transmission of mortality risk ratios, well studied in the Swedish population ‘Overkalix cohort’ [46]. Concerning IDDM2 type I diabetes, it has been shown that the susceptibility locus is associated with an allelic polymorphism (VNTR) at the insulin gene (*INS*): the alleles having 26 to 63 repeats (class I) predispose to type I diabetes disease in the homozygous state, while the alleles having from more than 140 repeats (class III) act as a dominant protective factor against the disease. However the study of a specific allele of class I (the *allele 814* having 42 repeats) has demonstrated that it did not predispose to the disease in the progeny as expected when the father carried the untransmitted class III alleles (the fathers were heterozygous: genotypically *allele 814*/*class III* for the *INS* gene) [43]. This result suggests that a sort of paramutation (the paramutagenic class III alleles *vs* the paramutable *814 allele*) acts in the inheritance of this disease.

## MOLECULAR BASIS OF PARAMUTATION

The mechanism involved in this self-propagating memory can be divided into two classes: the cis- acting signals physically associated with the gene that they regulate and the trans- acting signals. With the cis epigenetic signals belong DNA methylation or histones modification associated with a change in the chromatin structure and the consequent transcription machine accessibility on the gene promoter, although non-histone proteins also tightly associated with chromatin could be involved [47]. In contrast, trans epigenetic signals are maintained by soluble molecules such as transcription factors or small RNAs (sRNAs) acting in feedback loops of self regulation of own expression level [48, 49]. Recently it has been argued that prions could also represent a kind of epigenetic inheritance/paramutation-like phenomenon not based on nucleic acid but on the protein folding, resulting in different activity [40]. Prions are proteins which have different stable conformations: the native non-prion conformation usually is the more common but rarely, it may fold into a prion conformation that acquires the capability to catalyze the conformational conversion of the same (i.e. normal) native proteins through templating its prion structure. Of course these structural changes modify the native protein activity, causing the famous cases of infectious diseases mad cow disease and kuru. There is also the remarkable case of yeast (*S. cerevisiae*) where a prion protein is responsible for an advantageous dominant heritable trait modulated by environmental changes [50, 51]. Concerning the specific molecular mechanism involved in the basis of paramutation, so far three models have been proposed: a direct physical interaction between the paramutagenic and the paramutable alleles (pairing model), a gene inactivation mechanism mediated by RNA (small RNA model) and lastly a mix of both [33, 34, 36, 52].

It is known that DNA methylation plays an important role in paramutation, in fact usually the DNA of paramutagenic alleles is hypermethylated compared to their paramutable *alter-ego* alleles [53, 17, 24, 54, 55] although in some cases this association (paramutation/changes in DNA methylation) is not clear or does not appear at all, as in the case of the *kit* locus in mouse [41]. Also, repeated sequences**, **in direct as well as inverted orientation, (which seem to be associated to the silenced chromatin [56]) are present in most paramutation plant systems such as *r1 *[29], *p1* [24] and *b1* loci [57]: the repeated sequences can contain coding sequences as in the case of the *r1* locus or may be located upstream to the gene as in the cases of *b1 *and *p1 *genes. In the case of *b1*, seven copies of an 853 base pairs sequence are located about 100 kb from the coding region and they are associated to the paramutation onset (from *B-I* to *B’*) and paramutagenicity: in fact a neutral allele carrying a single copy, furthermore decreasing the number of repeats in *r1* and *b1,* causes a lowering in paramutagenicity [29, 57]. The transition from *B-I* to *B’* correlates with a hyper-methylation of tandem repeats and a differential sensitivity to DNaseI suggesting a different chromatin structure [57].

In the case of the *pl1* gene, repeated sequences have not yet been identified, although a complex allele named *pl-bol3* containing multiple *pl1* gene copies has been isolated from a Bolivian maize population [58] and it showed paramutation-like activity (R. Pilu, unpublished). Also, dosage effects caused by ploidy changes seem influence the paramutation as has been demonstrated in the tomato *sulf* locus [59] and in Arabidopsis active *hygromycin phosphotransferase* (*HPT*) transgene locus [60].

A potent tool to dissect the paramutation phenomenon consists in the isolation and study of the mutations that perturb the paramutation process: they can be subdivided roughly into two classes: (1) modifying the establishment of paramutation and (2) modifying the epigenetic memory [52]. Genetic screenings of mutagenized maize populations (carrying *B’* or *Pl’ *systems*) *using ethyl methanesulfonate have permitted the isolation of at least ten loci belonging to the first class named “*mediator of paramutation”* and to the second class named “*required to maintain repression”.* Out of the mutations isolated, all the genes cloned so far are involved in the RNA-directed transcriptional silencing: *mediator of paramutation1* (*mop1*) encoding for RNA-dependent RNA polymerase [61, 35], *mediator of paramutation2* (*mop2*) gene encoding for a second-largest subunit of plant-specific RNA polymerases IV and V [62], *required to maintain repression1* (*rmr1*) gene encoding for an SNF2-like ATPase, a chromatin-remodeling enzyme [63] and *required to maintain repression6* (*rmr6*) encoding the largest subunit of the plant specific DNA-dependent RNA polymerase [64]. In particular *mop1* is involved in the biogenesis of 24 nt siRNA and synthesis of dsRNA [65] as in the previously studied homologous orthologous *RDR2* Arabidopis gene [66]. Also in the *kit* paramutation system in mouse an involvement of siRNA has been demonstrated, in fact microinjecting RNA extracted from sperm or brain of mice with the white tail tips phenotype (carrying *Kit^tm1Alf^* allele) into fertilized wild mice eggs has been demonstrated to induce paramutation at the wild kit locus [41], resembling the result obtained in the experiment performed on *C. elegans* which led to the discovery of RNA interference (RNAi) [12] and may also be comparable to the maternal transmission of small RNA molecules called piwi-RNA (piRNAs) in *Drosophila melanogaster* [67]. Taken together, these recent findings demonstrate an essential role for RNAi processes in paramutation. The RNAi process includes the gene silencing effects of microRNAs (miRNAs) as well as silencing induced by foreign dsRNA: thus, paramutation and miRNA share in some way the same cellular machinery [68]. It is well known that DNA repeats are able to generate aberrant RNA (such as dsRNA inducing RNAi). However, in the case of *b1,* experimental data showed it was likely that the tandem repeats are not directly involved in the genesis of siRNAs but instead they are required as cis-signaling in the paramutation [69].

With the aim to isolate proteins involved in paramutation processes, the yeast one-hybrid technology has been used to identify the proteins binding to the repeated sequences present in most paramutagenic alleles. This strategy has been used successfully in the case of *b1* in which a CXC-domain protein CBBP (CXC domain *b1*-repeat binding protein) has been isolated sharing homology with some transposases able to bind *in vivo* and *in vitro* specifically a sequence within the tandem repeats of 853 bp inducing repressive chromatin states [70]. To confirm this finding, a transgenic maize overexpressing CBBP was created. In these plants we observed an induction of a silent state at the *b1* locus and this change was hereditable and the silent epiallele obtained (in the absence of a transgenic construct) was paramutagenic although with a reduced strength in comparison with *B’.* Furthermore CBBP forms multimers binding the *b1* tandem repeats suggesting a correlation between the strength of paramutation and the number of *b1* repeats and a possible trans interaction between chromosomes as observed in Drosophila in the case of transvection [71]. It is notable that CBBP mRNA levels are the same in the *B-I* and *B’* whilst the CBBP protein is only detectable in the *B’,* suggesting that a posttranscriptional control of CBBP is involved in the establishment of the *B’* state [70].

So far the relationship between RNAi machinery and CBBP is not clear but CBBP might be involved in same way in the chromatin modification complex as hypothesized for Drosophila CXC domain proteins [72]. Hence CBBP defines a new class of protein involved in gene silencing, not sharing similarity to the Arabidopsis RNAi silencing pathway [70]. Taking together all the data obtained so far using the best studied *b1* locus it is possible to speculate regarding a paramutation model: an increase of CBBP protein level (probably due to a stochastic posttranscriptional control) causes the onset of *B’* from *BI*, this state is maintained in the next generation by RNA and/or proteins signals associated with the *b1* repeats during mitosis and meiosis; in some way a pair trans interaction between *B’* and *B-I* repeats establishes the paramutation Fig. (**4**). Interestingly, another phenomenon involving RNAi-mediated heterochromatin in yeast and arabidopsis does not show paramutation capacity [73, 74] strengthening the idea that although RNA-induced silencing complex (RISC) and RNA-induced transcriptional silencing (RITS) are involved in the paramutation phenomenon, this last could represent a new system to propagate epigenetic information.

## SPECULATION ON THE MEANING OF PARAMUTATION

Paramutation may represent a rare “dull” deregulation of the system involved in the establishment and maintaining of chromatin state in a particular genome region defining the epigenetic state. Otherwise the biological systems where paramutation has been discovered have in common two characteristics: first the genes involved determined a phenotype easy to score by a simple visual inspection such as pigment [13, 28, 41] or by an easy colorimetric assay [17] or involving a serious disease [43]; second, all these traits are monogenic characters representing a small subset of the genes present in whole genome. These considerations lead us to suppose that paramutation phenomena could be more common than previously thought. In fact any paramutation phenomenon involving QTL would be hard to be find due to the small amount of phenotypic modification caused by a change in a single or a few genes expression level involved in the phenotypic complex trait. Thus several hypotheses regarding the functions of paramutation have been formulated, for example: the involvement in physiological systems evolved to control the expansion of sequences in the genome such as transposons and viruses able to expand in the genome across the generations [75], to regulate gene expression in polyploids, a function in inbreeding depression and in the corresponding hybrid vigor [8]. We can also speculate that paramutation could play an important role in the rapid transmission of particular epialleles in the populations in a way of course not predicted by the Hardy and Weinberg principle**. **Furthermore in the *r1* paramutation system it has been shown that environmental stimuli such as temperature and light can modify in an hereditable way the *r1* expression states, suggesting a Lamarckian-like behavior of this trait [76].

## CONCLUSION

Paramutation is associated in some way to siRNA biogenesis and in most cases to repeated sequences closely linked to the gene undergoing paramutation. Although it has been hypothesized for many years that repeated sequences were involved in the transcription of the aberrant RNA triggering an RNA-directed transcriptional silencing, a recent paper regarding the *B1 *paramutation phenomenon [70] suggests that these repeated sequences contain target sequences recognised by DNA binding proteins involved in the onset of silencing and correlated with paramutation capacity. So far the relationship between the siRNA pathway and the regulation of these proteins that are probably involved in the chromatin modification complex is not clear. Considering the increasing interest in epigenetic and paramutation-like phenomenon in recent years, we can foresee that the huge amount of data released, in particular genomics and transcriptomics data, will shed light on the spread and mechanism of this transmission of epigenetic information.

## Figures and Tables

**Fig. (1).**
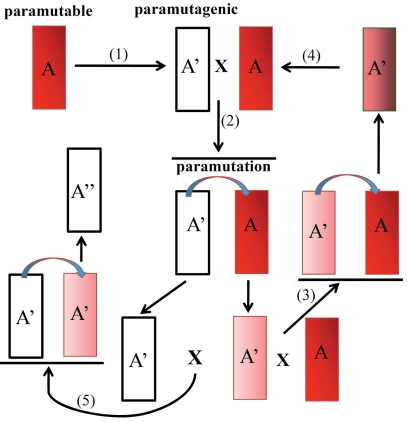
Scheme of classical paramutation phenomenon. Color intensity (from red = high expression to white = low expression) represents phenotypic expression of the A haplotype. A paramutable A allele undergoes spontaneous silencing inducing also paramutagenic activity (1). In the A’/A heterozygous (obtained by crossing individual carrying paramutagenic A*’* allele with the paramutable A allele) the haplotypes segregating in the offspring are both A’ (because A’ has paramuted A) although the new A’ allele is less silenced than the original one (2). If the A’ paramutated allele is crossed with a paramutable allele a “secondary paramutation” is observed in the progeny (3). If a paramutated A’ allele is not exposed again in trans with the original A’ allele in few generations it will come back to the A paramutable phenotype (4). Crossing again the paramutated A’ allele with the strongest paramutagenic A’ this will induce in the progeny the reinforcing of the silencing in the A’’ haplotype (5).

**Fig. (2).**
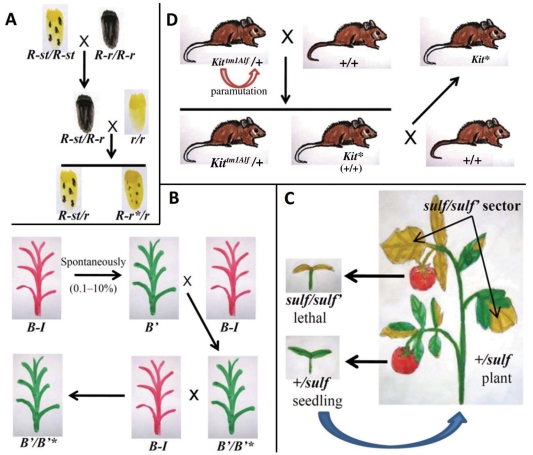
Pedigree of the most famous cases of paramutation described in plants and animals. Paramutation in maize at the *r1* locus (paramutable *R-r* and paramutagenic *R-st* alleles) involving the accumulation of anthocyanins in the maize seed (**A**) and in the whole plant in the case of *b1* locus (paramutable *B-I* and paramutagenic *B*’** alleles) (**B**). Paramutation in tomato at the *sulf* locus (paramutable + and paramutagenic *sulf* alleles) causes chlorophyll-deficient phenotype (yellow color) (**C**). In mouse paramutation at the *kit* locus (paramutable + and paramutagenic *Kit^tm1Alf^* alleles) confers white tail tips (**D**).

**Fig. (3).**
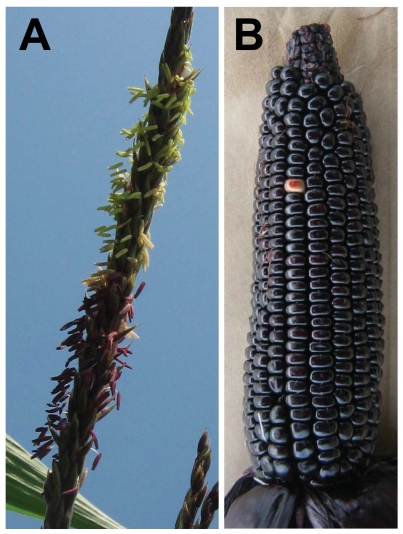
Spontaneous paramutation occurring at the *pl1* locus in maize. A sector of yellow anther on the tassel (**A**) and one weakly colored seed on the ear (**B**) are shown in a *B-I/B-I Pl-Rh/Pl-Rh* plant (genotype conferring a strong anthocyanin accumulation on the whole plant).

**Fig. (4).**
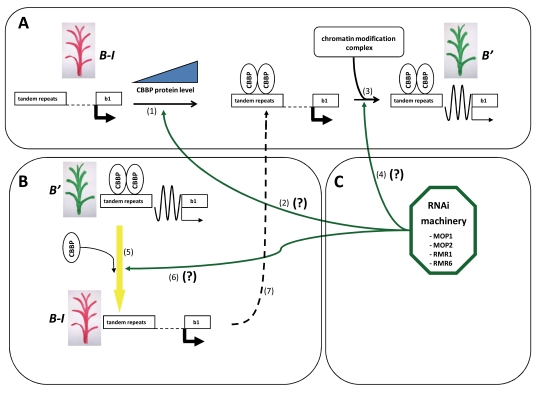
Paramutation model in the *b1* locus. In (**A**) is shown the spontaneous appearance of paramutagenic *B*’** from *B-I,* in (**B**) the *B*’** paramutation activity *vs* *B-I* and in (**C**) the four genes so far discovered involved in the RNAi machinery are indicated. The description of the model: (**A**) the *B-I* allele (red pigmented plant) is depicted by two boxes representing the seven tandem repeats and the *b1* gene, the two boxes are united by hyphens indicating an active conformation of chromatin in this DNA region. Marked black arrows starting from *b1* box represents the high transcription levels of *B-I* allele. An increase of CBBP protein level determines the binding of these proteins to the tandem repeats (1), in this step the RNAi machinery could also be involved (2). The CBBP proteins bonded to the tandem repeats in some way trigger the recruitment of the chromatin modification complex (3) which determines an hereditable silent conformation of chromatin structure (depicted by the sinusoid line between the two boxes) causing a strong decrease in *b1* transcription levels (depicted by the thin black arrow starting from *b1* box) and this new *b1* epiallele named *B*’** (green pigmented plant) acquires paramutagenic capacity. (**B**) When a *B-I allele* is exposed in trans with a *B’* paramutagenic allele (by crossing), an interaction (5) involving CBBP protein which binds the tandem repeats of *B-I* allele and perhaps a physical interaction between pairing genomic region on two chromosomes (indicated by the yellow arrow) and participation of RNAi machinery (4) cause the paramutation of *B-I* allele as described in A (7). In (**C**) are shown the proteins so far found involved in the maize paramutation: with the exception of CBBP are all implicated in the RNAi system: MOP1, MOP2, RMR1 and RMR6.
